# Accuracy of diagnostic tests for respiratory syncytial virus infection within a paediatric hospital population in Kilifi County, Kenya

**DOI:** 10.12688/wellcomeopenres.16067.1

**Published:** 2020-07-01

**Authors:** Marshal M. Mweu, Nickson Murunga, Juliet W. Otieno, D. James Nokes

**Affiliations:** 1 School of Public Health, College of Health Sciences, University of Nairobi, Nairobi, Kenya; 2 KEMRI-Wellcome Trust Research Programme, Kilifi, Kenya; 3 School of Life Sciences, University of Warwick, Coventry, UK

**Keywords:** Bayesian latent class analysis; Respiratory syncytial virus; Direct immunofluorescence test; PCR; Test accuracy; Hospitalised children

## Abstract

**Background:** Respiratory syncytial virus (RSV)-induced lower respiratory tract disease is a prominent cause of hospitalisation among children aged <5 years in developing countries. Accurate and rapid diagnostic tests are central to informing effective patient management and surveillance efforts geared towards quantifying RSV disease burden. This study sought to estimate the sensitivity (Se), specificity (Sp) (along with the associated factors) and predictive values of a direct immunofluorescence test (IFAT), and two real-time reverse transcription polymerase chain reaction (rRT-PCR) assays for RSV infection within a paediatric hospital population: a multiplex rRT-PCR (MPX) and Fast-Track Diagnostics ^®^ (FTD) Respiratory Pathogens 33 (Resp-33) rRT-PCR.

**Methods:** The study enlisted 1458 paediatrics aged ≤59 months admitted with acute respiratory illness at the Kilifi County Hospital between August 2011 and December 2013. A Bayesian latent class modelling framework was employed to infer the tests’ estimates based on the patients’ diagnostic data from the three tests.

**Results:** The tests posted statistically similar Se estimates: IFAT (93.7%, [90.7; 95.0]), FTD (97.8%, [94.6; 99.4]) and MPX (97.5%, [94.2; 99.3]). As for Sp, FTD registered a lower estimate (97.4%, [96.2; 98.2]) than MPX (99.7%, [99.0; 100.0]) but similar to IFAT (99.0%, [98.2; 99.6]). The negative and positive predictive values were strong (>91%) and closely mimicked the pattern given by the Se and Sp values respectively. None of the examined covariates (age, sex and pneumonia status) significantly influenced the accuracy of the tests.

**Conclusions:** The evaluation found little to choose between the three diagnostic tests. Nonetheless, with its relative affordability, the conventional IFAT continues to hold promise for use in patient care and surveillance activities for RSV infection within settings where children are hospitalised with severe acute respiratory illness.

## Introduction

Respiratory syncytial virus (RSV) is a leading cause of bronchiolitis and pneumonia globally, with an estimated 3.2 million hospital admissions and 59,600 in-hospital deaths being registered in children aged <5 years in 2015 ^1^. In Kilifi, Kenya’s coast, RSV is associated with seasonal epidemics characterised by high under-five hospitalisation rates ^2, 3^.

As RSV clinical presentation is indistinguishable from other acute respiratory infections, virologic testing of respiratory secretions is necessary to establish an RSV diagnosis ^4, 5^. Since 2002, longitudinal surveillance for RSV at the Kilifi County Hospital (KCH), seeking to strengthen the understanding of paediatric RSV epidemiology, has depended on a direct immunofluorescence test (IFAT) (RSV DFA kit, light Diagnostics ^TM^) as the standard diagnostic tool ^2, 6^. Of note, IFAT’s merits stem from its good performance (high sensitivity (Se) and specificity (Sp)) when used during peak RSV season, ease of use and interpretation, rapidity and affordability ^7^. Nevertheless, the test’s accuracy may vary depending on the virus strain targeted and sufficiency of the specimen ^8– 10^. Furthermore, the test’s Se may suffer especially in immunocompromised individuals and during RSV inter-epidemic periods when virus transmission is low ^11, 12^.

To enhance the detection of RSV, as of 2008, RSV surveillance at KCH has integrated the use of real-time reverse transcription polymerase chain reaction (rRT-PCR) assays, namely: a custom (in-house) multiplex rRT-PCR (MPX) and Fast-Track Diagnostics ^®^ (FTD) Respiratory Pathogens 33 (Resp-33) rRT-PCR ^6^. PCR assays possess superior Se to immunofluorescence tests, particularly during off-seasons, owing to their comparably lower viral detection limits ^13^. They afford further advantages in permitting the quantification of viral loads in samples and the detection of multiple respiratory viruses. However, owing to the tests’ costliness, their applicability in most resource-limited settings is restricted. Moreover, since viral particles may be detectable by the tests in healthy subjects, thereby compromising their Sp, the clinical usefulness of PCRs remains vague ^14^. An understanding of the performance of these RSV diagnostics in a clinical setting is valuable for informing clinical decision-making and supporting RSV-targeted genetic studies.

Conventionally, evaluation of the performance of RSV diagnostics has relied on the use of imperfect reference tests ^15– 18^ that may introduce bias to the accuracy estimates of index tests. Further, a call for alternative evaluation approaches is justified especially when interest lies in demonstrating the superiority of a novel tests over an existing reference standard ^19^. Bayesian latent class models (BLCMs) permit the quantification of test accuracy without knowledge on the true infection status of subjects ^20^.

When data are available on covariate factors believed to affect the characteristics of tests under evaluation, stratum-specific estimates are derivable and, arguably, afford greater utility than pooled estimates in inhomogeneous populations ^21^. Notably, Se and Sp are properties specific to a test; unlike predictive values, they do not provide information as to the probability that an individual has a certain disease considering their test status. Thus, the objective of the present study was to derive the Se and Sp together with the predictive values of IFAT, MPX and FTD tests for RSV infection within a paediatric hospital population, while assessing the effect of specific factors on the accuracy of the tests.

## Methods

### Study setting and population

As part of the existing long-term surveillance for RSV disease in children at KCH ^2^, the study enrolled paediatric patients aged ≤59 months admitted with syndromic pneumonia (as described by Hammitt *et al.* ^22^) between August 2011 and December 2013. Notably, KCH is the primary referral facility in Kilifi County admitting ~4000 paediatric patients annually. Here, for children aged <5 years, hospitalisation rates for RSV have been observed in the region of 271–317 admissions per 100,000 children annually ^2^. Additionally, RSV epidemics in Kilifi are strongly seasonal usually occurring between the months of November of one year and May of the next ^2, 23^.

### Sample collection

On admission, paired nasopharyngeal and oropharyngeal samples were collected and processed as previously described ^24^. The samples were stored in cooler boxes and delivered to the hospital’s microbiology laboratory for processing within 2 hours. At the lab, the samples were stored at 4°C until processed – usually within 48 hours of collection. The specimens were screened for the presence of RSV by IFAT, MPX and FTD tests. Aside from the samples, the patients’ sociodemographic characteristics (date of admission, sex, age, area of residence and presenting symptoms) were recorded.

### Ethical considerations

Prior to enlisting the paediatric patients into the study, written informed consent was obtained from their parents/guardians. Approval for the study was granted by the Kenya Medical Research Institute (KEMRI) Ethical Review Committee (SERU No. 3178 and SCC Nos. 1858 and 1526) and Oxford Tropical Ethical Review Committee, UK (60-90).

### Target condition

The infection (latent) status targeted by the three tests (IFAT, MPX and FTD) constitutes an RSV-laden respiratory sample carrying either the live virus or its antigens at any concentration.

### IFAT

A Light Diagnostics ^TM^ RSV DFA kit (Cat No. 3125, Chemicon, USA) was used to test for the presence of RSV in respiratory samples. The IFAT procedure was run as per manufacturer’s instructions. Briefly, 200 µl of specimen was used for slide preparation following a centrifugation process (Cytospin 3, Themo Shandon) at 1800 rpm for 10 min. A drop of Light Diagnostics RSV DFA reagent was added to the fixed cell preparation. The ready slide was then incubated in a moist chamber at 37°C for 30 min. Excess reagent was washed off in a fresh change of phosphate buffered saline, with the slide allowed to air dry at room temperature. After adding a drop of mounting fluid to the centre of each well, the stained specimen was examined with an epifluorescence microscope at ×200–500 magnification. An observation of apple-green fluorescence in one or more cells of the stained specimen constituted a positive result. 

### MPX assay

The MPX assay has been detailed elsewhere ^24^. Briefly, 140 µl of respiratory specimen was employed to extract RNA by Qiacube HT using an RNeasy extraction kit (Qiagen, Germany). The extracted RNA was subsequently tested for RSV (A and B) by a multiplex real-time PCR assay in triplex sets (Qiagen, ABI 7500 system). Samples yielding cycle threshold (Ct) values ≤35 were deemed positive for RSV.

### FTD assay

The procedure for the FTD assay is as previously described ^25^. Briefly, from a 400µl respiratory specimen, RNA was extracted using the NucliSENS easyMAG platform (bioMérieux, France). The extracted RNA was screened for RSV using the FTD Resp-33 PCR Kit. Quantitative PCR data were generated by creation of standard curves using serial dilutions of plasmid standards provided by FTD, with pathogen density being ascertained from the sample Ct values. As with the MPX assay, Ct values ≤35 were considered positive for RSV.

### Population classification

The KCH is nested within the Kilifi Health and Demographic Surveillance System (KHDSS) area in coastal Kenya – stretching about 891 km ^2^ and spanning a resident population of approximately 260,000 persons. Of note, KHDSS was established in 2000 with a view to monitoring vital statistics as well as, among other goals, quantifying the magnitude of significant local childhood ailments ^26^. Consequently, the diagnostic data represented a sample drawn from this single target population that informed the estimation of the Se and Sp of the tests.

### Statistical analysis

A Bayesian modelling framework was employed to derive the prevalence, Se and Sp estimates along with the predictive values of the tests. The BLCM was fitted in OpenBUGS software (v 3.2.2) ^27^ but called from R (v. 3.6.2) via the ‘ BRugs’ package (v0.9-0) ^28^. Importantly, the model design and reporting were guided by the standards for the reporting of diagnostic accuracy studies that use BLCMs (STARD-BLCM) ^29^. The Bayesian code is available as underlying data ^30^.

Essentially, BLCMs are premised on three key assumptions: (i) the target population should consist of two or more subpopulations with different prevalences, (ii) the Se and Sp of the index tests should be conditionally independent given the disease status and (iii) the Se and Sp of the tests should remain constant across the subpopulations ^31^. By definition, conditional independence implies that given a subject’s disease status, knowledge of results of one test does not alter the expectation about the result of a subsequent test ^32^. In our context, given an individual’s RSV infection status, the IFAT test was presumed to be conditionally independent to the two PCR assays considering the tests’ distinct targets: antigens versus genes. However, to allow for dependence between the two PCRs, we specified two conditional covariances between pairs of the Se and Sp ( *γ _Se_*, *γ _Sp_*) of the assays as detailed by Gardner *et al.* ^33^. Values of *γ _Se_* or *γ _Sp_* excluding zero denote significant dependence in Se or Sp estimates of the tests. To assess the separate effects of ‘sex’, ‘age’ (categorised into <6 months and ≥6 months ^34^) and ‘pneumonia status’ on the Se and Sp of the tests, the assumption on constancy of the test characteristics was relaxed by stratifying the single population into subpopulations specified by the covariate levels. As for pneumonia, a child’s presenting symptoms following admission were used to infer their pneumonia status (severe or very severe) as previously defined ^22^. The stratification permitted the estimation of stratum-specific tests estimates. Differences between the stratified estimates were tested using a Bayesian *P*-value.

Counts ( *O _m_*) of the different test combinations (e.g. +,+,+) were assumed to follow a multinomial distribution of the form:

$$O_{m}Se_{km}Sp_{km}P_{m}∼multinomial\left(prob_{m},n_{m}\right)$$

Where *Se _km_* and *Sp _km_* represent the respective test characteristics for test *k* ( *k* = 1,2,3) in subpopulation *m* and *P _m_* is the specific prevalence for the *m ^th^* ( *m* = 1,2) subpopulation. *Prob _m_* is a vector of probabilities of observing the different combinations of test results, and *n _m_* provides the number of subjects tested for the *m ^th^* subpopulation. For instance, in the 1 ^st^ subpopulation for a child testing positive to each of the three tests, incorporating dependence between the PCR assays, *prob _1_* is given by:

$$\begin{array}{l} prob_{1}=Pr\left({T_{1}}^{+}{T_{2}}^{+}{T_{3}}^{+}D^{+}\right)+Pr\left({T_{1}}^{+}{T_{2}}^{+}{T_{3}}^{+}D^{-}\right)\\ =Se_{11}\left(Se_{21}Se_{31}+\gamma_{se1}\right)P_{1}+\left[1-Sp_{11}\right]\left(\left[1-Sp_{21}\right]\left[1-Sp_{31}\right]+\gamma_{sp1}\right)\left[1-P_{1}\right] \end{array}$$

Since for each covariate, the two available subpopulations furnished only 14 degrees of freedom insufficient to compute the required 18 parameters (stratum-specific Se and Sp of the three tests, two subpopulation prevalences and four conditional covariances), prior information on at least four of the parameters was necessary to achieve model identifiability ^35^. As per available literature, the Se and Sp estimates of the standard IFAT test for paediatric RSV infection fall within the ranges: Se (77.8% - 95.1%) and Sp (82% - 99.6%) ^16, 17, 36– 40^. Accordingly, these test ranges were used to specify the uniform prior distributions for the IFAT test within the Bayesian model. For the remaining parameters, since no prior information was available, non-informative priors ( *beta*(1,1)) were utilised.

Positive and negative predictive values (PPV and NPV respectively) specific for test *k* and subpopulation *m* were derived as follows:

$$ppv=P_{m}Se_{km}/\left(P_{m}Se_{km}+\left[1-P_{m}\right]\left[1-Sp_{km}\right]\right)$$

$$npv=\left[1-P_{m}\right]Sp_{km}/\left(P_{m}\left[1-Se_{km}\right]+\left[1-P_{m}\right]Sp_{km}\right)$$

Additionally, a separate non-stratified model (disregarding differences in Se and Sp estimates across covariate levels) was built and the relative goodness of fit for the two model specifications compared using the Deviance Information Criterion (DIC) – model preferability being pegged on the smallness of the DIC value.

Two Markov Chain Monte Carlo chains were used to initiate the models. For each chain, a total of 70,000 iterations were run with the initial 20,000 discarded as the burn-in phase. Convergence of the chains was assessed by visual inspection of the time series plots of selected variables and the Gelman-Rubin diagnostic plots. The posterior distribution of the Se and Sp test estimates, subpopulation prevalences together with the predictive values were recorded as the median and associated 95% posterior credible intervals (PCI).

## Results

The cross-classified counts of the three-test results by covariate level are presented in Table 1. The data contained information from 1458 paediatric patients, of whom 43.8% ( *n* =638) were <6 months of age, 58.9% ( *n* =859) were male and 34.1% ( *n* =497) had very severe pneumonia.

**Table 1.** Cross-classified counts by stratum for IFAT, FTD and MPX tests for diagnosis of RSV infection among paediatric patients admitted with acute respiratory illness at KCH, Kenya during the period August 2011–December 2013.

Stratum	Tests outcomes combinations (IFAT; FTD; MPX)								Total (%)
	+++	++-	+-+	-++	+--	-+-	--+	---	
Single population	295	4	3	20	13	27	1	1095	1458 (100%)
Age									
<6mths	177	2	1	7	6	7	1	437	638 (43.8%)
≥6mths	118	2	2	13	7	20	0	658	820 (56.2%)
Sex									
Female	132	0	2	11	6	12	0	436	599 (41.1%)
Male	163	4	1	9	7	15	1	659	859 (58.9%)
Pneumonia status									
Severe	215	4	2	12	9	16	1	702	961 (65.9%)
Very severe	80	0	1	8	4	11	0	393	497 (34.1%)

The stratified estimates of Se and Sp for the three tests are displayed in Table 2. The estimated tests’ characteristics did not vary significantly by any of the examined factors as given by the Bayesian *P*-value. As the non-stratified model had better fit (DIC = 42.5) to the data compared to the covariate-specific models (DICs = 76.2; 72.8; 72.3), subsequent analyses were based on the former model.

**Table 2.** Stratified estimates of sensitivity and specificity of IFAT, FTD and MPX tests for paediatric RSV infection and a Bayesian *P*-value for the difference in the stratified estimates.

Test parameter ^a^	Covariate		Bayesian *P*-value ^b^
	Age		
	<6 months Estimate (95% PCI)	≥6 months Estimate (95% PCI)	
*Se _IFAT_*	94.3 (91.5; 95.1)	91.0 (84.8; 94.8)	0.89
*Se _FTD_*	98.2 (94.7; 99.8)	96.7 (91.5; 99.3)	0.75
*Se _MPX_*	97.6 (94.0; 99.5)	96.7 (91.2; 99.5)	0.64
*Sp _IFAT_*	98.8 (97.3; 99.6)	99.1 (98.0; 99.6)	0.37
*Sp _FTD_*	98.1 (96.4; 99.1)	96.7 (94.9; 97.9)	0.91
*Sp _MPX_*	99.5 (98.4; 100.0)	99.7 (98.5; 100.0)	0.39
	Sex		
	Female Estimate (95% PCI)	Male Estimate (95% PCI)	
*Se _IFAT_*	92.5 (87.5; 95.0)	93.8 (90.2; 95.1)	0.29
*Se _FTD_*	97.1 (92.5; 99.3)	98.2 (94.1; 99.8)	0.31
*Se _MPX_*	98.7 (94.2; 100.0)	96.2 (91.8; 98.7)	0.86
*Sp _IFAT_*	98.8 (97.4; 99.6)	99.1 (98.0; 99.6)	0.35
*Sp _FTD_*	96.9 (94.8; 98.3)	97.5 (96.1; 98.5)	0.27
*Sp _MPX_*	99.6 (98.1; 100.0)	99.7 (98.8; 100.0)	0.48
	Pneumonia status		
	Severe Estimate (95% PCI)	Very severe Estimate (95% PCI)	
*Se _IFAT_*	94.0 (90.9; 95.1)	91.3 (84.0; 94.9)	0.83
*Se _FTD_*	97.8 (94.2; 99.5)	96.9 (91.1; 99.5)	0.64
*Se _MPX_*	96.9 (93.1; 99.0)	98.3 (92.6; 99.9)	0.29
*Sp _IFAT_*	98.9 (97.8; 99.6)	99.0 (97.7; 99.6)	0.47
*Sp _FTD_*	97.5 (96.1; 98.5)	96.8 (94.6; 98.3)	0.75
*Sp _MPX_*	99.7 (98.8; 100.0)	99.6 (98.0; 100.0)	0.56

a Median estimates

b Value is significant if outside the interval 0.025; 0.975

Estimates of the Se and Sp of the RSV diagnostics together with their predictive values are displayed in Table 3. All the tests exhibited high (>93%) Se and Sp values. The Se estimates were statistically similar. Nonetheless, FTD registered a lower Sp (97.4%; 95% PCI [96.2; 98.2]) than MPX (99.7%; 95% PCI [99.0; 100.0]) but similar to the IFAT test (99.0%; 95% PCI [98.2; 99.6]). Consequently, FTD’s PPV estimate was similar to IFAT (96.5%; 95% PCI [93.5; 98.4]) but comparably lower (91.4%; 95% PCI [87.7; 94.2]) to that of MPX (99.1%; 95% PCI [96.3; 99.9]). The tests’ NPV estimates were high and numerically similar. The proportion of truly RSV-infected children amongst those hospitalised with acute respiratory illness was 22.2% (95% PCI [20.1; 24.5]). The two PCR assays demonstrated statistically significant conditional dependence.

**Table 3.** Estimates of prevalence, sensitivity and specificity of IFAT, FTD and MPX tests for paediatric RSV infection and their respective predictive values along with the conditional covariances for the dependence between FTD and MPX assays.

Parameter	Estimate (95% PCI)
*Se _IFAT_*	93.7 (90.7; 95.0)
*Se _FTD_*	97.8 (94.6; 99.4)
*Se _MPX_*	97.5 (94.2; 99.3)
*Sp _IFAT_*	99.0 (98.2; 99.6)
*Sp _FTD_*	97.4 (96.2; 98.2)
*Sp _MPX_*	99.7 (99.0; 100.0)
*P*	22.2 (20.1; 24.5)
*NPV _IFAT_*	98.2 (97.3; 98.7)
*NPV _FTD_*	99.4 (98.4; 99.8)
*NPV _MPX_*	99.3 (98.3; 99.8)
*PPV _IFAT_*	96.5 (93.5; 98.4)
*PPV _FTD_*	91.4 (87.7; 94.2)
*PPV _MPX_*	99.1 (96.3; 99.9)
*γse* ^a^	0.89 (0.01; 3.67)
*γsp* ^a^	0.13 (0.001; 0.83)

^a^Value is significant if the 95% PCI excludes zero.

## Discussion

We have estimated the accuracy and predictive values of IFAT, FTD and MPX for the diagnosis of RSV infection in hospitalised paediatric patients under a Bayesian paradigm. Arguably, this framework permits the quantification of accuracy of index tests devoid of misclassification errors inherent in diagnostic evaluations utilising imperfect reference standards ^41^. Thus, the derived estimates can be deemed readily extendable to similar populations of hospitalised children with severe respiratory illness. 

With observed disparities in nasopharyngeal RSV loads between ages ^42^, sexes ^43^ and disease severity classes ^44, 45^, it is plausible that performance of the RSV diagnostics may be affected by these covariates. This evaluation was conducted, but, as depicted in Table 2, the Se and Sp estimates of the three tests remained comparable across the examined covariate levels. This observation implies that the accuracy of any of the tests is unaffected by the age, sex and severity status of the presenting paediatric patient. Nevertheless, with advancing age (>5 years), owing to diminished viral titres in nasal secretions, the Se of most antigen-based assays (e.g. IFAT) may be undermined ^7^, thus precluding their utility in RSV diagnosis in the aforementioned population.

Of the sample of paediatric patients admitted with acute respiratory illness, 22.2% had an RSV infection – asserting the pathogen’s preponderance amongst pneumonic patients in the study setting ^23^. Correspondingly, the three tests recorded strong and similar Se estimates; although molecular assays, particularly for RNA viruses, may be affected by primer/probe mismatches which may impair their Se ^6^. The FTD assay revealed a lower Sp than MPX but similar to the IFAT test. This discordance in the Sp results between the two assays may be reflective of the non-Sp of the RSV matrix gene targeted by FTD as compared to MPX’s nucleoprotein gene target that permits the latter to effectively discriminate between RSV A and B viruses ^6^.

All the tests yielded high NPV and PPV values. As for PPVs, the FTD estimate was lower than MPX but comparable to IFAT – a pattern explained by the tests’ specificities. The strong confidence in both negative and positive test outcomes realised from these high predictive values is especially pivotal in patient management so as to: (1) establish the diagnosis of viral as opposed to bacterial cause of respiratory illness hence lessen unnecessary antibiotic prescriptions ^46^, (2) minimise supplementary testing such as blood tests and chest X-rays ^47^, (3) support cohorting of children to limit nosocomial spread within facilities ^48^ and (4) shorten the duration of hospital stay ^49^. Despite the observed good performance of the PCR diagnostics for RSV, their applicability in many resource-limited settings (outside research) is constrained attributable to their high costs and requirement of well-trained personnel. Accordingly, granted IFAT’s similar performance (to the PCR assays) coupled with its relative affordability, these attributes lend the test suitable for long-term use in resource-poor settings to foster patient care and surveillance activities aimed at quantifying RSV burden.

A noteworthy caution is that the estimates derived in the present study relate strictly to severely ill hospitalised children. Since patients presenting to outpatient facilities with less severe illness may, conceivably, harbour lower viral titres in respiratory secretions, generalisability of our findings to this population is not guaranteed.

## Conclusions

We have derived the Se and Sp and predictive values of IFAT, FTD and MPX for diagnosis of RSV infection in a paediatric population admitted with severe acute respiratory illness. The tests registered high (>93%) Se and Sp estimates, with correspondingly high NPV and PPV values. Notably, the tests’ characteristics were unaffected by age, sex or pneumonia status of the admitted patient. The strong accuracy recorded by the standard IFAT test renders it suitable for continued use in patient care and surveillance activities for RSV infection within populations of children hospitalised with severe respiratory illness.

## Data availability

### Underlying data

Since the raw dataset carries sensitive information on participants, it has been stored under restricted access. Access is available for researchers and can be applied for by placing a request to our Data Governance Committee at dgc@kemri-wellcome.org. The replication data and analysis scripts for this manuscript are available from the Harvard Dataverse.

Harvard Dataverse: Replication data for: Accuracy of diagnostic tests for respiratory syncytial virus infection within a paediatric hospital population in Kilifi County, Kenya. https://doi.org/10.7910/DVN/LWFP30 ^30^.

This project contains the following underlying data:

- BLCM_RSV_tests_code.R (R script for analysis)
- KCH_RSV_tests_data.tab (Analysis dataset)
- MweuMarshal_RSV_Diagnostics_Codebook.pdf (Data dictionary containing variable description and value labels)
- MweuMarshal_RSV_Diagnostics_Readme.txt (Readme file)

Data are available under the terms of the Creative Commons Zero “No rights reserved” data waiver (CC0 1.0 Public domain dedication).
