# Supplementary material for: Accuracy of diagnostic tests for respiratory syncytial virus infection within a paediatric hospital population in Kilifi County, Kenya
Source: Wellcome Open Res. 2020 Jul 1;5:155. [Version 1] doi: 10.12688/wellcomeopenres.16067.1 (PMC7499398; doi:10.12688/wellcomeopenres.16067.1)
Supplement: Peer review history

# Peer review: Reviewer response for version 1

**DOI:** 10.21956/wellcomeopenres.17625.r40231

**Published:** 2020-09-17

**Authors:** Oscar Ngesa

**Affiliations:** 1 Department of Mathematics, Statistics and Physical Sciences, Taita Taveta University, Taita Taveta, Kenya

This is a well written paper aimed at applying a Bayesian approach to estimate the sensitivity (Se), specificity (Sp) and predictive values of three selected tests for Respiratory syncytial virus (RSV) in the absence of a gold standard. In the statistical analysis, employing the Bayesian latent class models, the authors mention that some parameters did not have information and some vague priors were used (Uniform(0,1)/Beta(1,1)). However, the authors have not conducted any sensitivity analysis to evaluate the effect of changing the selected priors; this should be explored.

Is the work clearly and accurately presented and does it cite the current literature?

Yes

If applicable, is the statistical analysis and its interpretation appropriate?

Partly

Are all the source data underlying the results available to ensure full reproducibility?

Yes

Is the study design appropriate and is the work technically sound?

Yes

Are the conclusions drawn adequately supported by the results?

Yes

Are sufficient details of methods and analysis provided to allow replication by others?

Yes

Reviewer Expertise:

Statistical Modeling, Bayesian Analysis, Spatial Analysis, Resilience and Food Security Analysis.

I confirm that I have read this submission and believe that I have an appropriate level of expertise to confirm that it is of an acceptable scientific standard, however I have significant reservations, as outlined above.

# Peer review: Reviewer response for version 1

**DOI:** 10.21956/wellcomeopenres.17625.r40232

**Published:** 2020-09-08

**Authors:** Henry Mwambi

**Affiliations:** 1 School of Mathematics, Statistics and Computer Science, University of KwaZulu Natal, Pietermaritzburg, South Africa

The paper is well written and easy to read yet applying a novel statistical approach namely Bayesian Latent Class Models. The paper employs Bayesian estimation to calculate posterior estimates for diagnostic test measures: sensitivity, specificity, positive and negative predictive values. A detailed comparison of the immunofluorescence test (IFAT), and two assays namely MPX and FTD assay was carried accounting for the effect of three covariate namely age, sex and disease severity. The statistical analysis is well articulated and results clearly reported and tabulated. A little bit more discussion on how the latency comes bout in the model description may be more helpful even to a statistically inclined reader although it is understood this is not a statistical methodology paper. Also, the authors should give a clear statement of why a frequentist approach to the problem is not suitable.

Is the work clearly and accurately presented and does it cite the current literature?

Yes

If applicable, is the statistical analysis and its interpretation appropriate?

Yes

Are all the source data underlying the results available to ensure full reproducibility?

Partly

Is the study design appropriate and is the work technically sound?

Yes

Are the conclusions drawn adequately supported by the results?

Yes

Are sufficient details of methods and analysis provided to allow replication by others?

Yes

Reviewer Expertise:

Infectious disease modelling and Biostatistics methodology and application.

I confirm that I have read this submission and believe that I have an appropriate level of expertise to confirm that it is of an acceptable scientific standard.

# Peer review: Reviewer response for version 1

**DOI:** 10.21956/wellcomeopenres.17625.r39835

**Published:** 2020-08-19

**Authors:** Frank Coenjaerts

**Affiliations:** 1 Department of Medical Microbiology, University Medical Center Utrecht, Utrecht, The Netherlands

This is a very well-written paper designed to report on the comparison of three well-defined diagnostic methods to detect the respiratory syncytial virus (RSV) in clinical specimen. The work is particularly elegant due to the author's choice to investigate the possible influence of coveriates (age, sex, and pneumonia status) on the outcome of the different diagnostic methods. These covariates are often present in similar studies, but usually neglected.

 Major comments regarding the statistical analyses:

1. From a virological standpoint, the three key-assumptions required for the used Bayesian modelling framework are valid. In addition, the inclusion of the mentioned covariates offers important insights to the field.
2. From a merely technical standpoint, this referee is not qualified and highly recommends in-depth reviewing by a statistician or computational virologist.

Minor remark:

 In the Introduction the authors state that "Moreover, since viral particles may be detectable by the tests in healthy subjects, thereby compromising their Sp, the clinical usefulness of PCRs remains vague". Although authors are right that the clinical usefulness (if any) of detecting these low levels of virus "remains vaque", I do not agree that this "compromises their Sp". Sp, specificity, is an exact finding, clinical usefulness the interpretation thereof.

Is the work clearly and accurately presented and does it cite the current literature?

Yes

If applicable, is the statistical analysis and its interpretation appropriate?

I cannot comment. A qualified statistician is required.

Are all the source data underlying the results available to ensure full reproducibility?

Yes

Is the study design appropriate and is the work technically sound?

Yes

Are the conclusions drawn adequately supported by the results?

Yes

Are sufficient details of methods and analysis provided to allow replication by others?

Yes

Reviewer Expertise:

Clinical Virology; Virological Diagnostics.

I confirm that I have read this submission and believe that I have an appropriate level of expertise to confirm that it is of an acceptable scientific standard, however I have significant reservations, as outlined above.
